# Review of Three-Dimensional Handheld Photoacoustic and Ultrasound Imaging Systems and Their Applications

**DOI:** 10.3390/s23198149

**Published:** 2023-09-28

**Authors:** Changyeop Lee, Chulhong Kim, Byullee Park

**Affiliations:** 1 Department of Electrical Engineering, Convergence IT Engineering, Mechanical Engineering, Medical Science and Engineering, Graduate School of Artificial Intelligence, and Medical Device Innovation Center, Pohang University of Science and Technology, Pohang 37673, Republic of Korea; ckdduq0801@postech.ac.kr; 2 Department of Biophysics, Institute of Quantum Biophysics, Sungkyunkwan University, Suwon 16419, Republic of Korea

**Keywords:** photoacoustic imaging, ultrasound imaging, 3D handheld, clinical applications, biomedical studies

## Abstract

Photoacoustic (PA) imaging is a non-invasive biomedical imaging technique that combines the benefits of optics and acoustics to provide high-resolution structural and functional information. This review highlights the emergence of three-dimensional handheld PA imaging systems as a promising approach for various biomedical applications. These systems are classified into four techniques: direct imaging with 2D ultrasound (US) arrays, mechanical-scanning-based imaging with 1D US arrays, mirror-scanning-based imaging, and freehand-scanning-based imaging. A comprehensive overview of recent research in each imaging technique is provided, and potential solutions for system limitations are discussed. This review will serve as a valuable resource for researchers and practitioners interested in advancements and opportunities in three-dimensional handheld PA imaging technology.

## 1. Introduction

Photoacoustic (PA) imaging is a non-invasive biomedical imaging modality that has been gaining increasing attention due to its unique ability to provide high-resolution structural and functional information on various endogenous light absorbers without needing exogenous contrast agents [1,2,3,4,5]. This imaging technique is based on the PA effect [6], which occurs when a short-pulse laser irradiates a light-absorbing tissue, causing local thermal expansion and extraction to occur and subsequently generating ultrasound (US) waves. Collected US signals are reconstructed to PA images that combine beneficial features of both optics and acoustics, including high spatial resolution, deep tissue penetration, and high optical contrasts. Further, PA images can visualize multi-scale objects from cells to organs using the same endogenous light absorbers [7,8]. Moreover, compared to traditional medical imaging modalities such as magnetic resonance imaging (MRI), computed tomography (CT), and positron emission tomography (PET), PA imaging systems offer several advantages, including simpler system configuration, relatively lower cost, and the use of non-ionizing radiation [9,10]. These benefits have resulted in the emergence of PA imaging as a promising technique for various biomedical applications, including imaging of cancers, peripheral diseases, skin diseases, and hemorrhagic and ischemic diseases [11,12,13,14,15,16,17,18,19,20,21,22].

Two-dimensional (2D) cross-sectional PA imaging systems are widely used due to their relatively simple implementation by adding a laser to a typical US imaging system. Moreover, PA images can be acquired at the same location as conventional US images, thus facilitating clinical quantification analysis [23,24,25,26,27]. However, despite the advantages of 2D imaging, poor reproducibility performance can arise depending on the operator’s proficiency and system sensitivity, which can adversely affect accurate clinical analysis [28]. To address these issues, the development of three-dimensional (3D) PA imaging systems utilizing various scanning methods such as direct, mechanical, and mirror-based scanning has gained momentum. Direct scanning employs 2D hemispherical or matrix-shaped array US transducers (USTs) for real-time acquisition of volumetric PA images [29,30]. Mechanical scanning combines 1D array USTs of various shapes with a motorized stage to obtain large-area PA images, albeit at a relatively slower pace. Mirror-scanning-based 3D PA imaging utilizes single-element USTs with microelectromechanical systems (MEMS) or galvanometer scanners (GS), enabling high-resolution images with fast imaging speed, although it has a limited range of acquisition [31,32].

While the aforementioned fixed 3D PA imaging systems have demonstrated promising results, the inherent fixed nature of these systems can potentially limit the acquisition of PA images due to patient-specific factors or the specific anatomical location of the pathology under consideration [9,33,34,35]. Consequently, to expedite clinical translation, it becomes imperative to develop versatile 3D handheld PA imaging systems. Here, we aim to provide a detailed review of 3D handheld PA imaging systems. Our strategy involves classifying these systems into four scanning techniques—direct [30,36,37,38,39,40,41,42,43,44,45,46,47,48,49,50,51,52,53,54,55,56,57], mechanical [10,58,59,60,61,62], mirror [31,32,63,64,65,66,67], and freehand [50,67,68,69,70] scanning—and providing a comprehensive summary of the latest research corresponding to each technique. Freehand scanning involves image reconstruction through image processing techniques with or without additional aids such as position sensors and optical patterns. It is not limited by the UST type. Further, we discuss potential solutions to overcome 3D handheld PA imaging system limitations such as motion artifacts, anisotropic spatial resolution, and limited view artifacts. We believe that our review will provide valuable insights to researchers and practitioners in the field of 3D handheld PA imaging to uncover the latest developments and opportunities to advance this technology.

## 2. Classification of 3D Handheld PA Imaging Systems into Four Scanning Techniques

**Figure 1:**
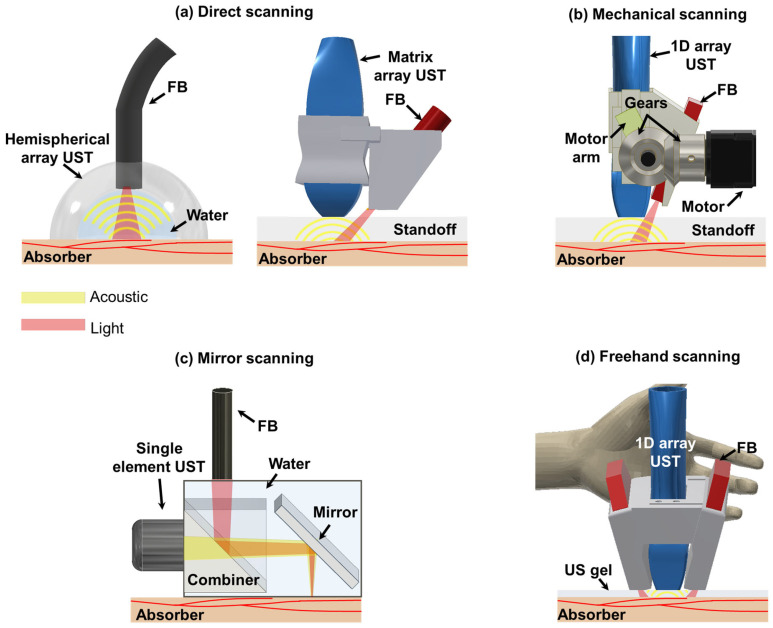
Schematics of 3D handheld PA imaging systems. (**a**) Direct scanning with 2D array UST-based 3D handheld PA imaging system. (**b**) Mechanical scanning with 1D array UST-based 3D handheld PA imaging system. (**c**) Mirror scanning with single-element UST-based 3D handheld PA imaging system. (**d**) Freehand scanning with 1D array UST-based 3D handheld PA imaging system. PA, photoacoustic; UST, ultrasound transducer; FB, fibers; LS, linear stage.

Three-dimensional handheld PA imaging systems can be categorized into four distinct groups based on the scanning mechanism employed: direct scanning, mechanical scanning, mirror-based scanning, and freehand scanning (Figure 1). Each of these systems possesses unique characteristics and limitations. Therefore, it is essential for researchers to comprehensively understand general features associated with each system to facilitate further advancements in this field.

Direct scanning involves utilization of 2D array USTs with various shapes such as hemispherical and matrix configurations [30,40,57]. These 2D array USTs enable the acquisition of 3D image data, facilitating direct generation of real-time volumetric PA images. Imaging systems based on 2D array USTs offer isotropic spatial resolutions in both lateral and elevational directions thanks to the arrangement of their transducer elements. Furthermore, a 2D hemispherical array UST provides optimal angular coverage for receiving spherical spreading PA signals [71]. This mitigates the effects of a limited view, thereby enhancing the spatial resolutions of obtained images. It is crucial to maintain coaxiality between US and light beams to achieve good signal-to-noise ratios (SNRs). To accomplish this, some imaging systems based on a 2D hemispherical array UST or certain matrix array USTs incorporate a hole in the center of the UST array, which allows light to be directed towards the US imaging plane [39,46,54].

Mechanical-scanning-based 3D handheld PA imaging systems mainly use 1D array USTs [58,59]. With the help of mechanical movements, 1D array USTs can collect scan data in the scanning direction. Three-dimensional image reconstruction is typically performed by stacking collected data in the scanning direction. Since 1D array USTs provide array elements in the lateral direction, beamforming can be implemented in the lateral direction. However, they do not provide array elements in the elevation direction. Thus, beamforming reconstruction cannot be performed, thereby providing anisotropic spatial resolutions. Although the synthetic aperture focusing technique (SAFT) can be applied as an alternative using scan data in the elevational direction [72], its effectiveness is low due to a tight elevation beam focus fixed by the US lens. Fiber bundles (FBs) are routinely used for light transmission. They are obliquely attached to the side of USTs using an adapter. To cross the US imaging plane and the light area irradiated obliquely, they typically use a stand-off such as water or a gel pad between the bottom of USTs and image targets [9,10,58].

Mirror-scanning-based 3D handheld PA imaging systems use single-element USTs with different mirrors (e.g., MEMS or GS) to ensure high imaging speed [31,32]. Spatial resolutions of these systems are generally superior to array-based imaging systems because they use a single element with high frequency USTs. Some of them can provide tight optical focusing to provide better lateral resolutions [73]. Conversely, they can provide shallower penetration depth than other systems. In particular, penetration depths of these systems providing optical focusing are limited to 1 mm, the mean optical path length of the biological tissue [74]. Thus, these systems are disadvantageous to use for clinical imaging [63]. To ensure coaxial configuration between light and acoustic beams, they typically use beam combiners [64,67].

Freehand scanning can be implemented with all UST types. Additional devices such as a global positioning system (GPS) and optical tracker are attached to UST bodies for recording the scanning location [69,70]. With scan data and the information of the scanning location, 3D image reconstruction is implemented. In addition, by analyzing similarity between successively acquired freehand scan images, position calibration can be performed without additional hardware units [50]. Furthermore, they provide flexibility to combine with different scanning-based 3D PA imaging systems [67]. Therefore, spatial resolutions, penetration depths, and coaxial configurations might vary depending on the UST type and scanning system.

These four scanning mechanisms provide useful criteria for distinguishing 3D handheld PA imaging systems. Their respective characteristics are summarized in Table 1.

### 2.1. Three-Dimensional Handheld PA Imaging Systems Using Direct Scanning

Direct 3D scanning is typically achieved using hemispherical or matrix-shaped 2D array USTs. In PA imaging, the PA wave propagates in a three-dimensional direction, which makes hemispherical 2D array USTs particularly effective for wide angular coverage, minimizing limited view artifacts and ensuring high SNRs [71]. Meanwhile, matrix-shaped 2D array USTs can be custom fabricated into smaller sizes, making them highly suitable for handheld applications [54,57]. Furthermore, their unique features make them suitable for use as wearable devices [55].

**Figure 2:**
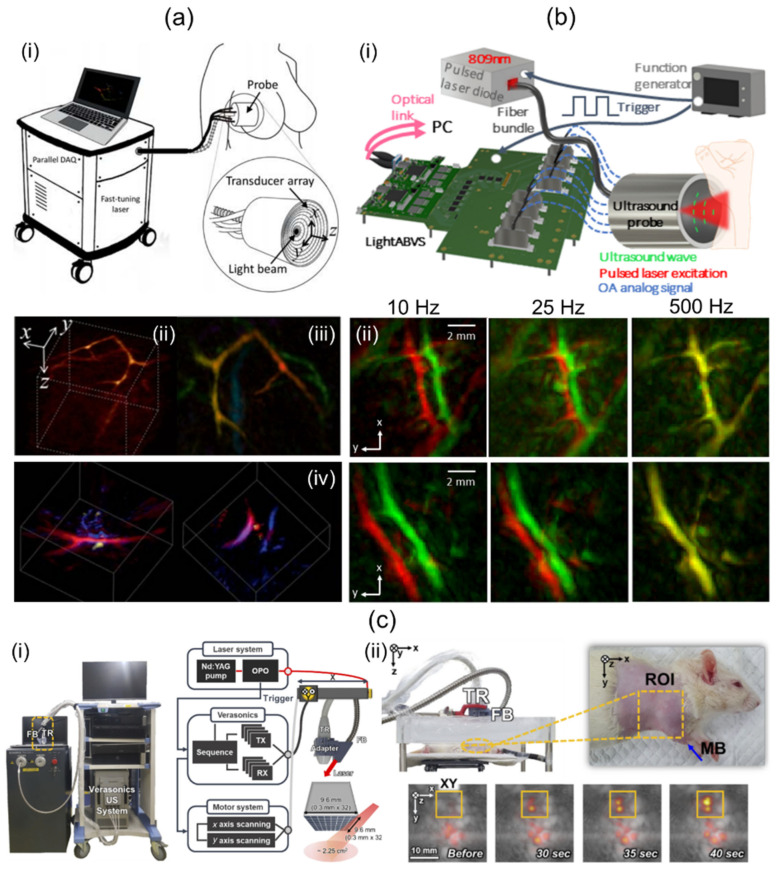
Direct-scanning-based 3D handheld PA imaging systems and their applications. (**a**) (**i**) Schematic diagram of a 2D hemispherical array transducer-based direct 3D imaging system and its clinical application. PA (**ii**) amplitude (**iii**) depth-encoded and (**iv**) sO_2_ images of human breasts. (**b**) (**i**) A cost-effective direct 3D handheld PA imaging system based on a 2D hemispherical array probe. (**ii**) In vivo human wrist PA MAP images acquired at various acquisition rates. Top and bottom rows indicate different regions around the wrist. (**c**) (**i**) Conventional 2D matrix array UST-based 3D PA/US imaging system. (**ii**) Photograph of the rat and in vivo PA/US MAP images after MB injection. ROI indicates the SLN area. PA, photoacoustic; US, ultrasound; MAP, maximum amplitude projection; SLN, sentinel lymph node; MB, methylene blue; sO_2_, oxygen saturation. Reprinted with permission from references [35,39,75].

Dea’n-Ben et al. [39] have introduced a 3D multispectral PA imaging system based on a 2D hemispherical array UST and visualized human breast with the system. They used an optical parametric oscillator (OPO) laser, a hemispherical shaped UST, and a custom designed data acquisition (DAQ) system (Figure 2a(i)). The OPO laser emitted short pulses (<10 ns) and was rapidly tuned over a wavelength range of 690 to 900 nm. The laser fluence and average power were less than 15 mJ/cm^2^ and 200 mW/cm^2^, respectively. The hemispherical US TR was composed of 256 piezocomposite elements arranged on a 40 mm radius hemispherical surface that covered a solid angle of 90°. The element was about 3 × 3 mm^2^. It provided 4 MHz of a center frequency with a 100% bandwidth. At the center of the TR, an 8 mm cylindrical cavity was formed. Light was delivered through the cavity using an FB. Data acquisition was conducted at 40 Msamples/s with the DAQ system. Single-wavelength volume imaging was performed at a rate of 10 Hz. For online image reconstruction, a back-projection algorithm accelerated with a graphics processing unit (GPU) was implemented. For accurate imaging reconstruction, 3D model-based image reconstruction was conducted offline. The spatial resolution was measured by imaging spherical-shaped geometry. It was about 0.2 mm. Spectral unmixing was performed using the least-square method. Unmixed PA oxy-hemoglobin (HbO2), deoxy-hemoglobin (Hb), and melanin images of human breast are shown in Figure 2a(ii).

Özsoy et al. [75] have demonstrated a cost-effective and compact 3D PA imaging system based on a 2D hemispherical array UST. Their study was performed using a low-cost diode laser, the hemispherical array UST, and an optical-link-based US acquisition platform [76] (Figure 2b(i)). The diode laser delivered a single-wavelength (809 nm) pulse with a length of about 40 ns. The lasers’ pulse repetition frequency (PRF) was up to 500 Hz. The output energy was measured to be about 0.65 mJ. The laser system was operated by a power supply and synchronized by a function generator. The size of the laser was 9 × 5.6 × 3.4 cm^3^, which was sufficiently smaller than OPO-based laser systems. The hemispherical array UST consisted of 256 piezoelectric composite elements with a 4 MHz center frequency and 100% bandwidth. It provided a solid angle of 0.59 π°. A total of 480 single-mode fibers were inserted through the central hole of the transducer and used to deliver the beam. The custom DAQ system had a total cost of approximately EUR 9.4. It offered 192 channels. Its measurements were 18 × 22.6 × 10 cm^3^. The DAQ system offered a 40 MHz sampling frequency with a 12-bit resolution. A total of 1000 sample data were collected for each channel, acquiring a region of 37.5 mm. Data were transmitted to a PC via 100G Ethernet with two 100 G Fireflies. Image reconstruction was performed with back projection [77]. The FOV of the reconstructed volume was 10 × 10 × 10 mm^3^. In vivo human wrist imaging was performed by freely scanning along a random path using the developed system. Two consecutively acquired PA images were overlaid in red and green as shown in Figure 2b(ii). The top and bottom rows represent different imaged regions around the wrist. As the image acquisition rate increases, the accuracy of spectral unmixing is expected to improve as two successive images cannot be distinguished.

Liu et al. [57] have presented a compact 3D handheld PA imaging system based on a custom-designed 2D matrix array UST. Their research was conducted using a custom fiber-connected laser, the custom fabricated 2D matrix array probe, and a DAQ. The laser delivering 10.7 mJ of pulse energy at the fiber output tip was 27 × 8 × 6 cm^3^ in size with a weight of 2 kg. The probe contained 72 piezoelectric elements with a size, center frequency, and bandwidth of 1 × 1 mm^2^, 2.25 MHz, and 65%, respectively. US elements were arranged to make a central hole (diameter: 2 mm). The light was delivered through the hole after passing through a collimator (NA: 0.23 in the air) and a micro condenser lens (diameter: 6 mm, f: 6 mm). The light was then homogenized with an optical diffusing gel pad to reshape a Gaussian-distributed beam in the imaging FOV. The dimensions and weight of the entire probe were <10 cm^3^ and 44 g, respectively. Then, 72-channel data for each volume were digitized by the DAQ, providing 128-channel 12-bit 1024 samples at 40 Msamples/s. Data acquisition timing was controlled by a programmed micro-control unit (MCU). Acquired volume data were reconstructed by the fast phase shift migration (PSM) method [56], which enabled real-time imaging. Spatial resolution quantification was implemented by imaging human hairs. Lateral and axial resolutions were measured to be 0.8 and 0.73 mm, respectively. The system took 0.1 sec of volumetric imaging time to scan an FOV of 10 × 10 × 10 mm^3^. The handheld feasibility of the system was demonstrated by imaging the cephalic vein of a human arm.

Although 3D handheld PA/US imaging using a conventional 2D matrix array UST has not been reported, 3D handheld operation is readily possible using stand-off or optical systems. Therefore, studies using 2D matrix array USTs will potentially lead to the development of 3D handheld PA/US imaging systems. Representatively, Kim et al. [35] have demonstrated a conventional 2D matrix array UST-based 3D PA/US imaging system. They used tunable laser, the 2D matrix array UST, an FB, a US imaging platform, and two motorized stages (Figure 2c(i)). The laser wavelength range and PRF were 660–1320 nm and 10 Hz, respectively. The 2D matrix array UST provides 1024 US elements with a center frequency of 3.3 MHz. For light transmission, FB was attached to the side of the 2D matrix array UST obliquely, and light was delivered to the US imaging plane. The US imaging platform offers 256 receive channels. Thus, four laser shots were required for one PA image. To obtain wide-field 3D images, they combined 2D matrix array UST with two motorized stages and implemented 2D raster scanning. For offline image processing, 3D filtered back projection and enveloped detection were conducted. To evaluate system performance, a 54 × 18 mm^2^ phantom containing 90 μm thick black absorbers was imaged with a total scan time of 30 min at a raster scan step size of 0.9 mm. Quantified axial and lateral resolutions were 0.76 and 2.8 mm, respectively. Further, they implemented in vivo rat sentinel lymph node (SLN) imaging after methylene blue injection (Figure 2c(ii)). Wang et al. [78] have showcased a 3D PA/US imaging system based on a conventional 2D matrix array UST. In their study, a tunable dye laser, the 2D matrix array UST, and a custom-built DAQ were used. The laser’s PRF and pulse duration were 10 Hz and 6.5 ns, respectively. The laser was coupled with bifurcated FB. The matrix array UST has 2500 US elements with a nominal bandwidth of 2–7 MHz. Thirty-six laser shots were required for the acquisition of one PA image. Image data transferred by the DAQ were then reconstructed with a 3D back projection algorithm [79] on a four-core central processing unit (CPU). This took 3 h. The reconstructed PA image’s volume was 2 × 2 × 2 cm^3^. For the benchmark of their system, gelatin phantom containing a human hair was imaged. Measured profiles in axial, lateral, and elevational directions were 0.84, 0.69, and 0.90 mm, respectively. In addition, in vivo mouse SLN imaging with methylene blue was performed for further verification (Table 2).

While 2D array USTs with a hemispherical shape offer advantages for PA imaging, their high cost, complex electronics, and high computational demands are cited as disadvantages. Additionally, their large element pitch sizes render them unsuitable for US imaging, limiting their use in clinical research. Conversely, matrix-shaped 2D array USTs are highly portable due to their light weight and small size. However, their suboptimal angular coverage for PA signal reception results in poor spatial resolution and limited view artifacts.

### 2.2. Three-Dimensional Handheld PA Imaging Systems Using Mechanical Scanning

Several 3D handheld PA imaging systems utilizing mechanical scanning methods and various types of USTs have been developed. Typically, these systems employ one- to three-axis motorized stages for 3D scanning. They are relatively inexpensive, requiring simple electronic devices. In this chapter, we will introduce two such systems: one based on a 1D linear array UST and another based on a single-element UST [10,58,59,62].

**Figure 3:**
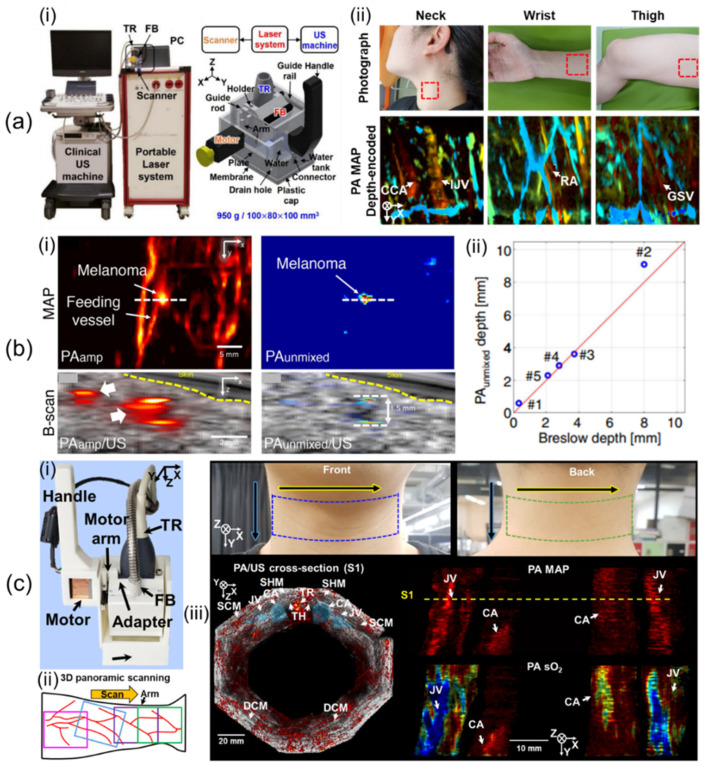
Three-dimensional clinical handheld PA/US imaging systems based on 1D linear array UST with mechanical scanning and their applications. (**a**) (**i**) Photograph of a mechanical-scanning-based 3D handheld PA/US imaging system using a 1D linear array UST. (**ii**) In vivo human imaging of various human bodies. (**b**) (**i**) US, PA amplitude, and unmixed PA melanin images of metastasized melanoma. (**ii**) Graph of unmixed PA melanin depth vs. histopathological depth. (**c**) (**i**) Updated 3D clinical handheld PA/US imager. (**ii**) Schematic of 3D panoramic scanning. (**iii**) Three-dimensional panoramic imaging of the human neck. PA, photoacoustic; UST, ultrasound transducer. Reprinted with permission from references [10,58,59].

Lee et al. [58] have developed a 1D linear array UST-based 3D handheld PA/US imaging system that uses a lightweight and compact motor whose scanning method is controlled by a non-linear scanning mechanism. In their study, they used a portable OPO laser with fast pulse tuning, a conventional clinical US machine, the 1D linear array UST, and a custom-built scanner (Figure 3a(i)). The laser delivered light at pulse energies of 7.5 and 6.1 mJ/cm^2^ at 797 and 850 nm, respectively, with a 10 Hz PRF. With 64 receive channels, the US machine was an FDA-approved US system that offered a programmable environment. The UST had an 8.5 MHz central frequency and a 62% bandwidth at −6 dB. The scanner included the guide rail, handle, guide rod, plate, connector, drain hole, plastic cap, FB, holder, UST, motor, arm, water, water tank, and membrane. The FB was integrated into the holder with the UST. It was inserted at 15° for efficient light transmission. The motor arm moved the holder to drive the UST in the scanning direction after receiving a laser trigger signal. The movement was managed with a non-linear scanning method (i.e., the scotch yoke mechanism) that changed linear motion into rotational movement and vice versa, allowing the use of a small, lightweight motor (170 g) instead of bulky, heavy motor stages. They used a 3 cm deep water tank to avoid PA reflection artifacts within 3 cm of the axial region. Polyvinyl chloride (PVC) membranes with a thickness of 0.2 mm were attached to the water tank to seal the water. Dimensions and weight of the scanner were 100 × 80 × 100 mm^3^ and 950 g, respectively. The maximum FOV was 40 × 38 mm^2^, and the maximum scan time for single-wavelength imaging was 20.0 s. Acquired volume data were reconstructed by Fourier-domain beamforming [80]. They measured spatial resolutions by quantifying the full width at half maximum (FWHM) of black threads having a thickness of 90 μm. The calculated axial and lateral resolutions were 191 μm and 799 μm, respectively. To confirm the usefulness of the handheld system for human body imaging, they implemented human in vivo imaging of the wrist, neck, and thigh (Figure 3a(ii)).

Using the developed 3D handheld imaging system, Park et al. [59] have conducted a clinical study of various types of cutaneous melanomas. They used multiple wavelengths of 700, 756, 797, 866, and 900 nm. The FOV was scaled to 31 × 38 mm^2^, resulting in a scan time of 57 sec. They recruited six patients who had in situ, nodular, acral lentiginous, and metastasis types of melanomas. US/PA/PA unmixed melanin images of metastasized melanoma were acquired as shown in Figure 3b(i). They compared unmixed PA melanin depth and histopathological depth and confirmed that the two depths agreed well within a mean absolute error of 0.36 mm (Figure 3b(ii)). Further, they measured a maximum PA penetration depth of 9.1 mm in nodular melanoma.

Lee et al. [10] have showcased updated 3D clinical handheld PA/US imaging scanner and systems. The 3D handheld imager weighs 600 g and measures 70 × 62 × 110 mm^3^, increasing handheld usability (Figure 3c(i)). The updated system improved SNRs of the system by an average of 11 dB over the previous system using a transparent solid US gel pad with similar attenuation coefficients to water. The previous system could not immediately check 3D image results, making it difficult to filter out motion-contaminated images. Through imaging system updates, PA/US maximum amplitude projection (MAP) preview images considered as 3D images were provided online on the US machine to help select data. Offline 3D panoramic scanning was performed to provide super wide-field images of the human body (Figure 3c(ii)). An updated 3D PHOVIS, providing six-degree mosaic stitching, was used for image position correction. Twelve panoramic scans were performed along the perimeter of the human neck for system evaluation (Figure 3c(iii)). The total scan time was 601.2 s, and the corrected image FOV was 129 × 120 mm^2^. Hemoglobin oxygen saturation (sO_2_) levels of the carotid artery and jugular vein were quantified to be 97 ± 8.2% and 84 ± 12.8%, respectively.

Bost et al. [62] have presented a single-element UST with a two-axis linear motorized stage-based 3D handheld PA/US imaging system. In their study, a solid laser, a custom probe, and a digitizer were used. The solid laser produced 532 and 1024 nm pulses with a PRF of 1 kHz and a pulse length of 1.5 ns. The maximum pulse energies at 523 and 1024 nm were 37 and 77 μJ, respectively. The probe integrated a 35 MHz single-element UST with 100% bandwidth, a custom-designed fiber bundle, a two-axis linear stage, and a waterproof polyvinylchloride plastisol membrane for acoustic coupling. The number and geometry of fibers were effectively designed with Monte Carlo simulation. Data were acquired at a sampling rate of 200 MSamples/s. For volumetric US and PA imaging, scan times were 1.5 min and 4 min, respectively, for an area of 9.6 × 9.6 mm^2^. US and PA lateral resolutions were 86 μm and 93 μm, respectively. A visualized B-scan superimposed US/PA images of a human scapula where the PA vascular network was identified. Furthermore, they acquired a volume-rendered image of a mouse with a subcutaneous tumor (Table 3).

Three-dimensional handheld imaging systems using mechanical scanning methods can be implemented with relatively simple devices while still providing acceptable image quality. However, these systems are susceptible to motor noises and vulnerable to motion contamination. Furthermore, the inclusion of motorized parts can make the entire scanner bulky and heavy, which can make handheld operation challenging.

### 2.3. Three-Dimensional Handheld PA Imaging Systems Using Mirror Scanning

Mechanical scanning is limited in speed due to physical movement of the scanner [81,82]. Additionally, the use of relatively bulky and heavy motor systems adversely affects handheld operation. To overcome these problems, optical scanning (e.g., MEMS- and galvanometer-based mirror scanners) was used to develop single-element UST-based 3D PA imaging systems.

**Figure 4:**
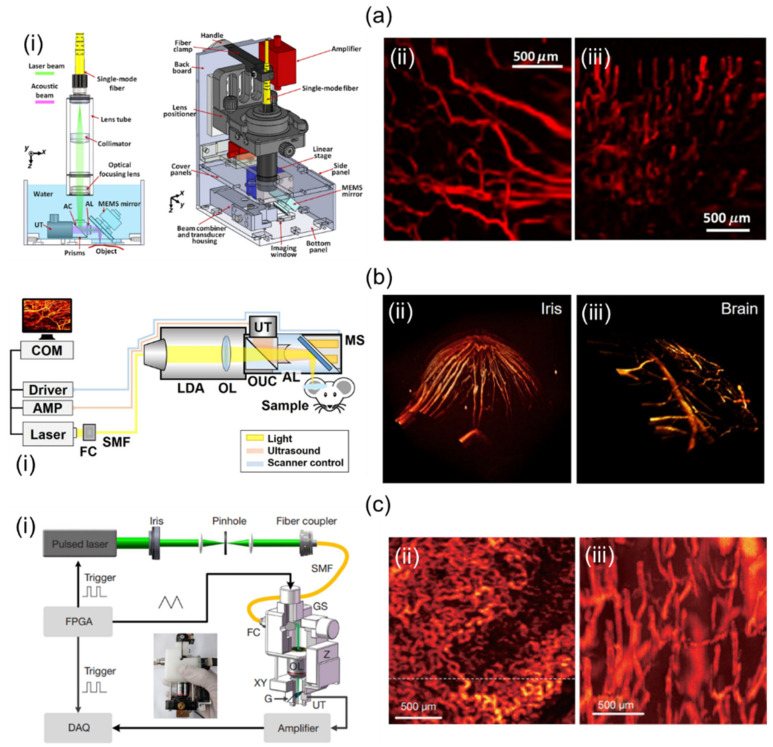
Mirror-scanning-based 3D handheld PA imaging systems and their applications. (**a**) (**i**) Schematic of a single-element UST and 2-axis MEMS-based 3D PA imaging system. In vivo PA vessel visualizations of (**ii**) a mouse ear and (**iii**) a human cuticle. (**b**) (**i**) Schematic diagram of a 2-axis MEMS and single-element UST-based miniaturized 3D handheld imaging system. In vivo PA volume rendered images of a mouse (**ii**) iris and (**iii**) brain. (**c**) Schematic diagram of a compact single-element UST-based 3D portable imaging system scanning with a galvanometer. In vivo PA MAP visualizations of (**ii**) the wattle of a Leghorn rooster and (**iii**) the lower lip of a human. MEMS, microelectromechanical systems; PA, photoacoustic; MAP, maximum amplitude projection. Reprinted with permission from references [31,32,64].

Lin et al. [64] have demonstrated a volumetric PA imaging system implemented using a two-axis MEMS and a single-element UST. For that study, they utilized a fiber laser, a custom handheld probe, and a DAQ. The laser pulse length at 532 nm was 5 nm, and the PRF was 88 kHz. The probe consisted of a single-mode fiber (SMF) for delivering the light, optical lenses for focusing laser beams into an opto-acoustic combiner, prisms for ensuring opto-acoustic coaxial alignment, the MEMS for reflecting focused laser beams and generated PA signals, a 50 MHz center frequency UST for detecting reflected PA signals, an optical correction lens for calibrating prism-induced aberration, and a motorized stage for correcting the location of the MEMS mirror (Figure 4a(i)). Laser pulses synchronized the MEMS scanner and the DAQ system. PA signal data were collected by the DAQ with a sampling frequency of 250 Msamples/s. PA volume imaging was implemented at a repetition rate of 2 Hz. The overall dimension and imaging FOV of the probe were 80 × 115 × 150 mm^3^ and 2.5 × 2.0 × 0.5 mm^3^, respectively. Resolution measurements of the system were performed in lateral and axial directions, resulting in 5 µm and 26 µm, respectively. To test the developed 3D handheld imager, a mouse ear (Figure 4a(ii)) and a human cuticle (Figure 4a(iii)) were imaged.

Park et al. [32] have developed a two-axis MEMS and single-element UST-based miniaturized system without using a mechanical stage. A diode laser, an SMF, a custom-designed 3D probe, and DAQ were used in their study. The 532 nm diode laser provided a 50 kHz PRF. It was coupled with optical fiber. The probe integrated light delivery assembly (LDA), objective lens (OL), opto-ultrasound combiner (OUC), the MEMS, an acoustic lens (AL), and a UST (Figure 4b(i)). After the light was delivered from the LDA, it was focused by the OL and delivered to the OUC containing prisms and silicon fluid. The delivered light was reflected by the MEMS scanner. It then illuminated objects that could absorb light. Generated PA signals were reflected from the MEMS and at prisms in OUC. Reflected PA signals were collected at the UST with a central frequency of 50 MHz. The AL attached to the right side of the OUC was used to increase SNRs. The DAQ was used to create trigger signals for operating the laser and the digitizer. At the same time, the DAQ generated sinusoidal and sawtooth waves to control the two-axis MEMS scanner. PA volumetric imaging was implemented at a PRF of 0.05 Hz. The probe was 17 mm in diameter with a weight of 162 g. It provided a maximum FOV of 2.8 × 2 mm^2^. Lateral and axial resolutions were 12 µm and 30 µm, respectively. To confirm the feasibility of the system, they conducted in vivo mouse imaging in various parts such as the iris and brain, as shown in Figure 4b(ii,iii).

Zhang et al. [31] presented a 2D GS and single-element UST-based 3D handheld system (Figure 4c(i)). Their study was performed using a pulsed laser, a custom-made probe, and a DAQ card. The laser offered a maximum PRF of 10 kHz at 532 nm. It was connected to an SMF (diameter: 4 μm) via a fiber coupler (FC). The light was delivered to a collimator mounted on the side of the probe. The collimated light was scanned with the GS and focused onto a transparent glass with an OL. Generated PA signals were reflected off the 45° tilted glass (G) and delivered to the UST with a center frequency of 15 MHz. The optical focus was adjusted using the probe’s XY- and Z-direction stages. The DAQ card provided a sampling rate of 200 MSamples/s. The 3D imaging took 16 sec. The imaging FOV was 2 × 2 mm^2^. Lateral and axial resolutions were quantified with the phantom’s sharp blade and carbon rod. The results were 8.9 µm and 113 μm, respectively. Using this system, they conducted in vivo rooster wattle and human lip imaging experiments, as shown in Figure 4c(ii,iii).

Qin et al. [65] have presented a dual-modality 3D handheld imaging system capable of PA and optical coherence tomography (OCT) imaging using lasers, a 2D MEMS, a miniaturized flat UST, and OCT units. For PA imaging, a pulsed laser transmitted light with a high repetition rate of 10 kHz and a pulse duration of 8 ns. For OCT imaging, a diode laser delivered light that provided a center wavelength of 839.8 nm with a FWHM of 51.8 nm. The light for PA and OCT imaging used the same optical path and was reflected by the MEMS mirror, respectively, to scan targets. The dual-modality 3D handheld scanner measured 65 × 30 × 18 mm^3^, providing an effective FOV of 2 × 2 mm^2^. Their lateral and axial PA resolutions were quantified to 3.7 μm and 120 μm, respectively, while lateral and axial OCT resolutions were quantified to 5.6 μm and 7.3 μm, respectively (Table 4).

Optical-scanning-based 3D PA probes provide high-quality PA images with fast acquisition and high handheld usability by eliminating motors and associated systems. However, theses probes have limitations such as small FOVs and shallow penetration depths, which are unfavorable for clinical PA imaging.

### 2.4. Three-Dimensional Handheld PA Imaging Systems Using Freehand Scanning

Three-dimensional handheld PA imaging systems utilizing freehand scanning methods have been developed using various types of USTs. This section aims to showcase a range of 3D freehand scanning systems that utilize different aids, including a GPS, an optical pattern, and an optical tracker. Additionally, we will explore freehand scanning imaging systems combined with other scanning-based imaging systems such as mirror-scanning-based and direct-scanning-based imaging systems.

**Figure 5:**
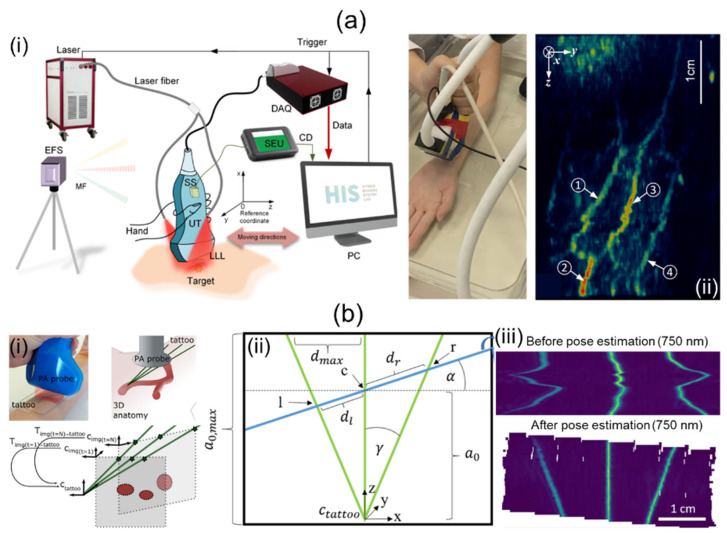
Freehand-scanning-based 3D handheld PA imaging systems and their applications. (**a**) (**i**) Schematic description of a free-scanning-based 3D handheld PA imaging system using a GPS sensor. (**ii**) In vivo 3D free-scan imaging of a human arm. (**b**) (**i**) Conceptual explanation of a 3D freehand scan imaging system using an optical tattoo. (**ii**) Schematic geometry for the tattoo. l, c, and r represent intersections of a free-scan image and the optical tattoo. (**iii**) Original and repositioned PA images acquired by freehand scanning. PA, photoacoustic; UT, ultrasound transducer; GPS, global positioning system. Reprinted with permission from references [69,70].

Jiang et al. [69] have proposed a 3D freehand PA tomography (PAT) imaging system using a GPS sensor. For their research, they utilized an OPO laser and a laser providing high power, a linear UST, a DAQ card, and a 3D GPS (Figure 5a(i)). The PRF and wavelength ranges of the OPO laser were 10 Hz and 690–950 nm, respectively. The high-power laser delivered a 1064 nm wavelength at a 10 Hz PRF. The energy fluence was less than 20 mJ/cm^2^. The linear UST provided a center frequency of 7.5 MHz with 73% bandwidth. The DAQ system sampled data on 128 channels at a rate of 40 Msamples/s. The GPS consisted of a system electronics unit (SEU), a standard sensor (SS), and an electromagnetic field source (EFS). The SS was attached to the US probe as shown in Figure 5a(i). It collected six degrees of freedom (DOF) data at a sampling rate of 120 Hz for each B-mode image. The 6-DOF data contained three translational (x, y, z) and three rotational (α, β, γ) data. Obtained 6-DOF data were transmitted to a PC via the SEU. Imaging positions were accurately tracked within a magnetic field generated by the EFS. GPS calculation errors for translational and rotational motions were 0.2% and 0.139%, respectively. They used the sensor within a translation resolution of 0.0254 mm and an angular resolution of 0.002° for 3D PAT. Free-scan data were acquired at scan speeds of 60–240 mm/min with scan intervals of 0.1–0.4 mm. After PA data collection, a back-projection beamformer was applied for 2D image reconstruction. Three-dimensional PAT was implemented as pixel nearest-neighbor-based fast-dot projection using 6-DOF coordinate data [83,84]. Measured lateral and elevational resolutions were 237.4 and 333.4 µm, respectively. The application of free-scan 3D PAT on a human wrist is shown in Figure 5a(ii).

Holzwarth et al. [70] have presented a 3D handheld imaging system using freehand scanning with a 1D concave array UST. They used a tunable ND:YAG laser, a multispectral opto-acoustic tomography (MSOT) Acuity Echo research system, and a custom optical pattern. The laser provided a spectral range of 660–1300 nm, with pulse energy, durations, and an emission rate of 30 mJ, 4–10 ns, and 25 Hz, respectively. The concave UST was 80 mm in diameter. It provided a central frequency of 4 MHz with 256 individual elements. The optical pattern was engraved on a transparent foil and filled with cyan ink. The pattern was attached to regions of interest (ROIs). Free-scan data were acquired within these ROIs. As shown in Figure 5b(i), the trident optical pattern representing the three green lines gave three points corresponding to each probe position in each free scan. They obtained a transformation matrix using probe positions and geometry (Figure 5b(ii)). Based on 2D images and the transformation matrix, 3D volume compounding was implemented [85]. Three-dimensional free-scan images before and after position calibration are shown in Figure 5b(iii).

Fournelle et al. [68] have demonstrated a 3D freehand scan imaging system using an optical tracker and a commercial 1D linear array UST. This study was performed using a solid-state ND:YAG laser, a wavelength tunable OPO laser, the linear array UST, a custom US platform, an optical tracking system, and an FB. Frame rates for the ND:YAG and OPO lasers were 20 and 10 frames/sec, respectively. Light emitted from these laser systems was transmitted through the FB. Due to the opening angle (22°) of the FB, the transmitted light formed a rectangular shape measured 2 × 20 mm^2^. The laser fluence was 10 mJ/cm^2^ at 1064 nm. The center frequency and pitch size of the UST were 7.5 MHz and 300 µm, respectively. The US platform digitized 128-channel data at a sampling rate of 80 MSamples/s. The optical tracker provided the position and orientation of each B-mode image with a root-mean-square error (0.3 mm3). An adaptive delay-and-sum beamformer was applied for reconstructing B-mode US and PA images. The reconstruction was accelerated with multi-core processors and parallel graphics processors. Reconstructed images were placed in 3D space using position and orientation information obtained from the optical tracker and corrected with a calibration matrix. Resolution characterization of the system was achieved by imaging the tip of a steel needle. Measured FWHMs in lateral and elevational directions were 600 µm and 1100 µm, respectively. To confirm the availability of the 3D freehand approach for humans, PA/US imaging was performed for a human hand. The total number of image frames acquired for the volume was 150, resulting in a scan time of 15 s.

Chen et al. [67] have showcased a 3D freehand scanning probe based on a resonant-galvo scanner and a single-element UST. The resonant mirror in the probe generated a periodic magnetic field to rotate the reflector at a rate of 1228 Hz. The galvo scanner was attached to the resonant mirror for slow-axis scanning. Taking advantage of the system’s high scanning speed (C-scan rate, 5–10 Hz), they applied simultaneous localization and mapping called SLAM. The SLAM performed feature point extraction between consecutive scan images using the speeded-up robust features (SURF) method and scale-invariant feature transform (SIFT) method [86,87,88,89]. Points were then used to calibrate positions of consecutive images. They demonstrated an extended FOV image of the mouse brain with the developed system and processing method. The expanded FOV was 8.3–13 times larger than the original FOV of ~1.7 × 5 mm^2^.

Knauer et al. [50] have presented a free-scan method using a hemispherical 3D handheld scanner to extend a limited FOV using acquired volumetric images. Acquired volume images with the hemispherical probe were spatially compounded. Fourier-based orientation and position correction were then applied. For translation (t_x_, t_y_, t_z_) and rotation (θ_x_, θ_y_, θ_z_) corrections, a phase correlation [90] and PROPELLER [91] were used, respectively. Volume areas that overlapped with each other by less than approximately 85% were used to apply the position correction algorithm. To validate their algorithm, they imaged a human palm. The extended volume FOV was 50 × 70 × 15 mm^3^, which was larger than the single volume image FOV (Table 5).

Freehand-scanning-based 3D PA imaging systems are implemented with many types of UST. They also offer flexibility to combine with mirror- or direct-scanning-based 3D PA imaging systems. Combined scanning methods can overcome limitations of small FOV systems. However, they can be severely affected by various motion artifacts, making spectroscopic PA analysis difficult.

## 3. Discussion and Conclusions

In Section 2 of this paper, we classified and provided detailed explanations of four scanning techniques (i.e., direct, mechanical, mirror-based, and freehand scanning) for 3D handheld PA imaging. Subsequently, in Section 2.1, Section 2.2, Section 2.3 and Section 2.4 we introduced recent research on 3D handheld PA imaging corresponding to these four scanning techniques. As mentioned earlier, 3D handheld PA imaging holds great potential for widespread applications, including both preclinical and clinical applications. Despite its active development and utilization, three major limitations need to be overcome: motion artifacts, anisotropic spatial resolution, and limited view artifacts. In the following sections, we will delve into these three limitations in greater detail.

**Figure 6:**
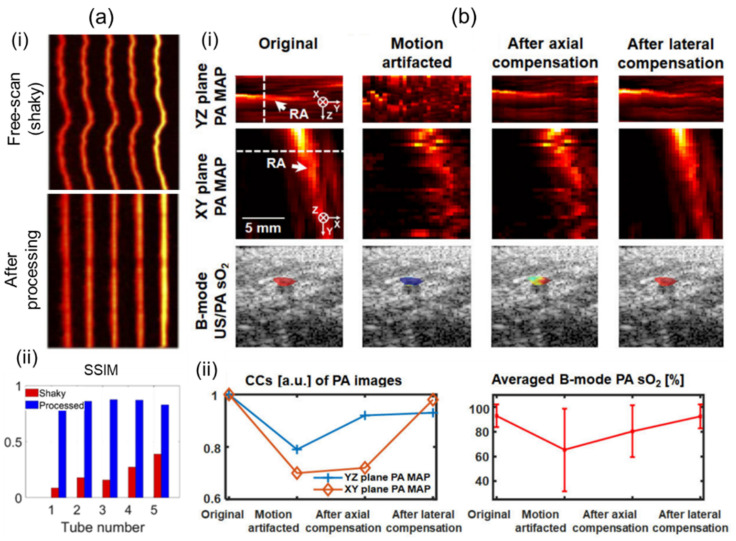
Motion compensations applied to 3D handheld imaging systems. (**a**) (**i**) Before and after motion-corrected PA MAP images. Motion contamination occurred in the freehand scan. (**ii**) Comparison of SSIM before and after motion correction. (**b**) (**i**) PA MAP and B-mode US/PA sO_2_ images before and after motion compensation. (**ii**) Motion compensation validation charts. Degrees of recovery of structural and functional information were measured. PA, photoacoustic; MAP, maximum amplitude projection; US, ultrasound; SSIM, the structural similarity index; sO_2_, hemoglobin oxygen saturation. Reprinted with permission from references [60,92].

Motion artifacts can occur in all types of scanning-based 3D imaging systems. Artifacts can degrade structural information. They can also deteriorate functional information such as sO_2_ due to pixel-by-pixel spectral unmixing calculations [60]. For clinical translation, 3D imaging systems should mitigate motion contaminations to ensure accurate imaging and quantifications. Mozaffarzadeh et al. [92] have showcased motion-corrected freehand scanned PA images using the modality independent neighborhood descriptor (MIND), which is based on self-similarity [93]. By applying the MIND algorithm, they corrected a motion-contaminated phantom image as shown in Figure 6a(i). After correction, the structural similarity index (SSIM) was greatly enhanced (Figure 6a(ii)). Yoon et al. [60] have proposed a 3D motion-correction method using a motor-scanning-based 3D imaging system. They applied US motion compensations by maximizing structural similarities of subsequently acquired US skin profiles. PA motion correction was implemented using the corrected US information. The effectiveness of motion compensation was verified through in vivo human wrist imaging (Figure 6b(i)). Structural similarities were measured by quantifying cross-correlations of each method. Significant improvements were confirmed after motion corrections. Further, inaccuracy of the spectral unmixing was greatly reduced after calibrations (Figure 6b(ii)).

**Figure 7:**
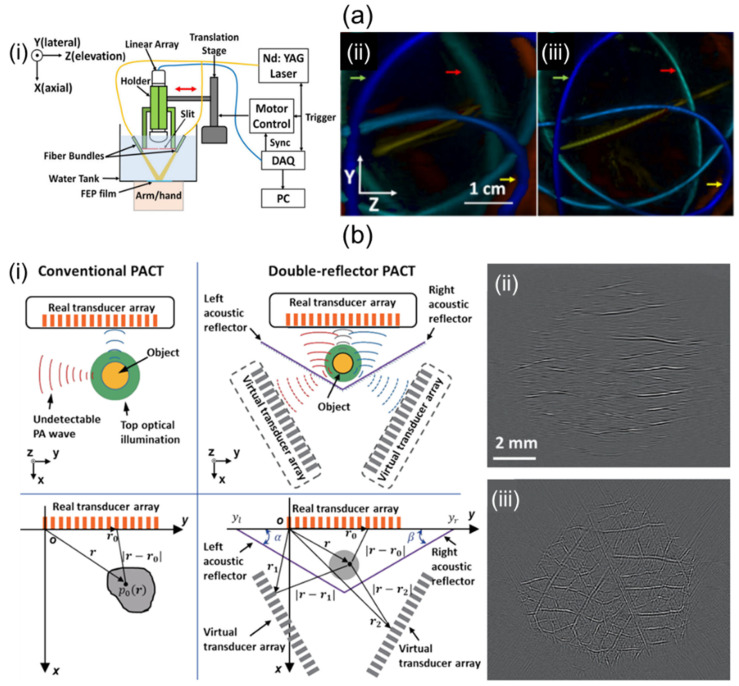
Potential ways to improve performance of 1D linear array UST-based 3D portable imaging systems. (**a**) (**i**) Schematic explanation of a slit-enabled PACT system. (**ii**) Conventional and (**iii**) slit-applied tube phantom depth-encoded PA images. (**b**) (**i**) Conceptual schematic of conventional and double-reflector PACT. PA leaf skeleton images of (**ii**) conventional and (**iii**) double-reflector application. The double-reflector PACT restored the vascular network of the leaf skeleton. PACT, photoacoustic computed tomography; UST, ultrasound transducer. Reprinted with permission from references [94,96].

It is known that 1D linear array UST-based 3D imaging systems suffer from low elevation resolution due to their narrow elevation beamwidth and limited view artifacts caused by a narrow aperture size. To overcome poor elevational resolution, Wang et al. [94] have proposed a system using stainless steel at the focal point of a 1D linear UST as shown in Figure 7a(i). The system diffracted PA waves to create wide signal receptions in the elevational direction [95]. They effectively applied the beamformer in the elevation direction with sufficiently overlapping signal areas. Results of depth-encoded tube phantom images before and after slit application are shown in Figure 7a(ii,iii). The elevation resolution of the system with slit was 640 μm, which was superior to the 1500 μm elevation resolution of the system without the slit.

To mitigate limited-view artifacts, solid angle coverage of a UST should be at least >π. Li et al. [96] have demonstrated a 1D linear array UST-based imaging system that applies two planar acoustic reflectors to virtually increase the detection view angle. The two reflectors were located at an angle of 120° relative to each other (Figure 7b(i)). The system using an increased detection view angle of 240° successfully recovered the vascular network image of the leaf skeleton (Figure 7b(iii)) compared to the original one (Figure 7b(ii)).

Recent advancements in silicon-photonics technology have led to the development of on-chip optical US sensors [97,98]. These sensors offer several advantages over traditional piezoelectric US sensors. Unlike piezoelectric sensors, which often sacrifice sensitivity when miniaturized, optical US sensors exhibit high sensitivity and broadband detection capabilities while maintaining a compact size. Furthermore, there have been recent breakthroughs in achieving parallel interrogation of these on-chip optical US sensors [99]. These innovations open up exciting possibilities for the creation of ultracompact 3D handheld imaging systems. In addition, multispectral imaging, real-time capabilities, hybrid modalities [100], and AI integration [101] hold great promise for improving future 3D handheld PA imaging systems. These systems can unlock new opportunities for patient care, early disease detection and image-guided interventions [102], ultimately enhancing our understanding and management of various medical conditions.

## Figures and Tables

**Table 1:** General features of 3D clinical handheld imaging systems.

Scanning Mechanism	Direct [30,39]	Mechanical [10,58]	Mirror [32,64]	Freehand
UST type	2D array	1D array	Single element	All types
Lateral resolution (μm)	200	592–799	5–12	*
Penetration depth (mm)	<30	<30	<1	*
Coaxial configuration unit	Hole	Standoff	Beam combiner	*

*: Depends on types of USTs and combined scan systems. UST, ultrasound transducer.

**Table 2:** Specifications of direct-scanning-based 3D handheld PA/US imaging systems.

Author		Dea’n-Ben et al. [39]	Özsoy et al. [75]	Liu et al. [57]	Kim et al. [35]	Wang et al. [78]
Laser	Wavelength	660–900 nm	809 nm	-	660–1320 nm	650 nm
	Pulse duration	<10 ns	40 ns	-	-	6.5 ns
	PRF	-	~500 Hz	-	10 Hz	10 Hz
	Light delivery	Fiber	Fiber	Fiber	Fiber	Fiber
Probe	Scanning aids	-	-	2 motor stages	-	-
	Scanning type	Direct	Direct	Direct	Direct + mechanical	Direct
	UST type	2D hemispherical	2D hemispherical	2D matrix	2D matrix	2D matrix
	# of elements	256	256	72	1024	2500
	Center frequency	4 MHz	4 MHz	2.25 MHz	3.3 MHz	-
	Bandwidth	100%	100%	65%	-	-
	Resolution	S: 200 μm	-	A, L: 730, 800 μm	A, L: 760, 2800 μm	A, L, E: 840, 690, 900 μm
	Scan time	0.1 s	0.002 s	0.1 s	-	3 h
	FOV	-	10 × 10 × 10 mm^3^	10 × 10 × 10 mm^3^	10 × 10 mm^2^	20 × 20 × 20 mm^3^
	Weight	-	-	44 g	-	-
	Dimension	-	-	<10 cm^3^	-	-
Data acquisition/ processing system	Platform	Custom DAQ	Custom DAQ	SonixDAQ	Verasonics	Custom DAQ
	Sampling rate	40 MHz	40 MHz	40 MHz	-	-

S, A, L, E: spatial, axial, lateral, and elevational resolutions, respectively.

**Table 3:** Specifications of mechanical-scanning-based 3D handheld PA/US imaging systems.

Author		Lee et al. [58]	Park et al. [59]	Lee et al. [10]	Bost et al. [62]
Laser	Wavelength	690–950 nm	690–950 nm	690–950 nm	532/1024 nm
	Pulse duration	5 ns	5 ns	5 ns	1.5 ns
	PRF	10 Hz	10 Hz	10 Hz	1 kHz
	Light delivery	Fiber	Fiber	Fiber	Fiber
Probe	Scanning aids	1 motor	1 motor	1 motor	2 motor stages
	Scanning type	Mechanical	Mechanical	Mechanical	Mechanical
	UST type	1D linear	1D linear	1D linear	Single element
	# of elements	128	128	128	1
	Center frequency	8.5 MHz	8.5 MHz	8.5 MHz	35 MHz
	Bandwidth	62%	62%	62%	100%
	Resolution	A, L: 191, 799 μm	A, L: 200, 1000 μm	A, L, E: 195, 592, 1976 μm	L: 93 μm
	Scan time	20 s	11.4 s	16.7 s	4 min
	FOV	40 × 38 mm^2^	31 × 38 mm^2^	25 × 38 mm^2^	9.6 × 9.6 mm^2^
	Weight	950 g	950 g	600 g	-
	Dimension	100 × 80 × 100 mm^3^	100 × 80 × 100 mm^3^	70 × 62 × 110 mm3	-
Data acquisition/ processing system	Platform	EC-12R	EC-12R	EC-12R	AMI US/OA platform
	Sampling rate	40 MHz	40 MHz	40 MHz	200 MHz

A, L, and E: axial, lateral, and elevational resolutions, respectively.

**Table 4:** Specifications of mirror-scanning-based 3D handheld PA/US imaging systems.

Author		Lin et al. [64]	Park et al. [32]	Zhang et al. [31]	Qin et al. [65]
Laser	Wavelength	532 nm	532 nm	532 nm	532 nm
	Pulse duration	5 ns	-	-	8 ns
	PRF	88 kHz	50 kHz	10 kHz	10 kHz
	Light delivery	Fiber	Fiber	Fiber	Fiber
Probe	Scanning aids	2D MEMS	2D MEMS	2D galvo	2D MEMS
	Scanning type	Mirror	Mirror	Mirror	Mirror
	UST type	Single element	Single element	Single element	Single element
	# of elements	1	1	1	1
	Center frequency	50 MHz	50 MHz	15 MHz	10 MHz
	Bandwidth	-	-	-	60%
	Resolution	A, L: 26, 5 μm	A, L: 30, 12 μm	A, L: 113, 9 μm	A, L: 120, 3.7 μm
	Scan time	0.5 s	20 s	16 s	-
	FOV	2.5 × 2.0 × 0.5 mm^3^	2.8 × 2 mm^2^	2 × 2 mm^2^	2 × 2 mm^2^
	Weight	-	162 g	-	-
	Dimension	80 × 115 × 150 mm^3^	12 cm	-	65 × 30 × 18 mm^3^
Data acquisition/ processing system	Platform	ATS9350	NI PCIe-6321	A DAQ card	NI PCI-5122
	Sampling rate	250 MHz	-	200 MHz	100 MHz

A and L: axial and lateral resolutions, respectively.

**Table 5:** Specifications of freehand-scanning-based 3D handheld PA/US imaging systems.

Author		Jiang et al. [69]	Holzwarth et al. [70]	Fournelle et al. [68]	Chen et al. [67]	Knauer et al. [50]
Laser	Wavelength	690 nm	660–1300 nm	532/1024 nm	532/588 nm	680–950 nm
	Pulse duration	-	4–10 ns	3–10 ns	-	-
	PRF	10 Hz	25 Hz	10–20 Hz	500 kHz	10 Hz
	Light delivery	Fiber	Fiber	Fiber	Fiber	Fiber
Probe	Scanning aids	GPS sensor	Optical pattern	Optical tracker	Resonant galvo	-
	Scanning type	Freehand	Freehand	Freehand	Freehand + mirror	Freehand + direct
	UST type	1D linear	1D concave	1D linear	Single element	2D hemispherical
	# Of elements	128	256	128	1	256
	Center frequency	7.5 MHz	4 MHz	7.5 MHz	-	4 MHz
	Bandwidth	73%	-	-	-	100%
	Resolution	L, E: 237, 333 μm	-	L, E: 600, 1100 μm	A, L: 39, 6 μm	S: 200 μm
	Scan time	240 mm/min	-	15 s	0.1–0.2 s	-
	FOV	45 × 38 × 38 mm^3^	-	-	1.7 × 5 mm^2^	50 × 70 × 15 mm^3^
	Weight	-	-	-	158 g	-
	Dimension	-	-	-	59 × 30 × 44 mm^3^	-
Data acquisition/ processing system	Platform	A DAQ card	MSOT Acuity Echo	DiPhAS	-	Custom DAQ
	Sampling rate	40 MHz	-	80 MHz	-	40 MHz

S, A, L, and E: spatial, axial, lateral, and elevational resolutions, respectively.
